# The distribution of fake Australian vaccine digital certificates on an alt-tech platform

**DOI:** 10.1007/s12117-022-09466-x

**Published:** 2022-10-27

**Authors:** Andrew Childs

**Affiliations:** Griffith Criminology Institute, Griffith University, Brisbane, QLD Australia

**Keywords:** Vaccine certificates, Cybercrime, Illicit markets, Online communities, Alt-tech platforms, Affordances

## Abstract

This paper provides the first exploration of the online distribution of fake Australian COVID-19 vaccine certificates. Drawing on a collection of 2589 posts between five distributors and their community members on the alt-tech platform *Gab*, this study gathers key insights into the mechanics of illicit vaccine certificate distribution. The qualitative findings in this research demonstrate the various motivations and binding ideologies that underpinned this illicit distribution (e.g. anti-vaccine and anti-surveillance motivations); the unique cybercultural aspects of this online illicit network (e.g. ‘crowdsourcing’ the creation of fake vaccine passes); and how the online community was used to share information on the risks of engaging in this illicit service, setting the appropriate contexts of using fake vaccine passes, and the evasion of guardians in offline settings. Implications for future research in cybercrime, illicit networks, and organised crime in digital spaces are discussed.

## Introduction

On the 11th of March 2020 the World Health Organisation declared that a novel coronavirus (SARS Cov-2) outbreak was a global pandemic (Cucinotta and Vaneli 2020). Following on from these events a combination of forces including the uncertainty of a new disease, its lethality, and sharp rises in daily case numbers meant that many of the world’s largest economies (e.g. China, USA, UK) halted as stay-at-home lockdown measures were implemented (Allen 2022; Joffe 2021; Ryan 2021). In the following years since the initial COVID-19 outbreak, even amid fluctuating surges of more contagious variants, countries have seemingly begun to shift towards an outlook on how to best accommodate COVID-19 in daily life. Worldwide efforts in COVID-19 vaccination distribution, in conjunction with “*fervent desires to return to normalcy*” (Hall and Studdert 2021), provided an impetus for a new public health approach characterised by vaccine mandates and certificates. Although some countries have achieved high vaccination rates, public perceptions of vaccines have somewhat hampered efforts and an abundance of psychological literature points to how a lack of perceived efficacy, a lack of trust in health officials, and low fears of the virus itself as markers for explaining this hesitancy (Ebrahimi et al. 2021; Gerretsen et al. 2021; Nazli et al. 2022). Some countries (e.g. India, various African countries) have also experienced issues such as fake vaccines (e.g. containing distilled water or saline solutions) (Aborode et al. 2021; Choudhary et al. 2021).

As of the beginning of 2022, vaccine mandates and accompanying digital certificates to verify an individual’s vaccination status against COVID-19 have been widely implemented (Milthani et al. 2022). Although the concrete manifestation of these certificates vary country-by-country, which itself is a product of the World Health Organisation’s (2021) suggestion that it is up to member states to determine the best approach, vaccine certificates typically contain some key characteristics including: the vaccine type (e.g. Pfizer, AstraZeneca, Moderna), the lot numbers for the vaccination, and a visual indication that an individual’s vaccination status is up-to-date (e.g. a green tick) (Milthani et al. 2022). The rationale for such measures is that public health restrictions should be tailored to risk and a gradual release of restriction can avoid further harsh lockdown measures. Furthermore, this method allows countries to reinstate normal economic conditions (e.g. restaurants, retail, tourism) while still attempting to limit the spread of COVID-19 (Milthani et al. 2022; Sharif et al. 2021). Vaccine mandates and accompanying certificates can also potentially provide an impetus for many individuals to get vaccinated. For example, in Mills and Ruttenauer’s (2022) examination of vaccine certification in six countries (Denmark, Israel, France, Germany, and Switzerland) the authors found that mandates and certification measures led to increased vaccination rates in the days prior to implementation. The use of vaccination passports has thus been seen as integral in managing the next phase of coronavirus where the world is seeking a return to normal functioning.

There has, however, been significant challenges in the roll-out of vaccination certificates, most prominently including the lack of attendance to cybersecurity risks and issues with data governance to protect an individual’s privacy (Gstrein 2021; Milthani et al. 2022). Another issue, the primary focus of this paper, is the emergence of fraudulent vaccination certificates. Media attention on the uptake of fake vaccine certificates has been reported in Australia (Trigger 2022), New Zealand (Arnold 2022), France (Ambrose 2022), and the United Kingdom (Raj 2021), but there is currently no academic research exploring this illicit product, the development of illicit networks for this service, and how fake vaccine certificates are distributed among individuals. Situated alongside growing attention on how COVID-19 shaped organised crime opportunities (Djordjevic and Dobovsek 2020; Maher and Wyatt 2021), the current study is interested in the development of organised crime and changing nature of illicit markets during COVID-19.

This study provides the first comprehensive exploration of the online distribution of fake vaccine certificates. In doing so, I focus on an Australian context as one country that implemented strict vaccine mandates. The Australian states and territories’ responses to COVID-19 initially resembled a ‘zero covid’ approach, encompassing targeted snap lockdowns to reduce the spread of coronavirus as quick as possible. The rollout of vaccines, in conjunction with smartphone applications that assisted individuals to check-in to venues to facilitate contact tracing, meant that public health policy then shifted away from the ‘zero covid’ approach. Here, states and territories embraced mandates and differed only slightly in terms of how the mandates were implemented and governed. While initially mandates were only for those working in what were deemed high-risk settings (e.g. healthcare workers), by December 2021, states and territories had begun enforcing vaccine mandates. Public venues and events (e.g. hospitality venues, government venues such as libraries and museums, sports stadiums, gyms) were only open for those who were vaccinated and mandatory COVID-19 vaccination requirements were also extended to numerous other occupations as employers became responsible for ensuring their workforce were vaccinated. Throughout 2022, all states and territories have engaged in a process of slowly relaxing vaccine requirements^1^.

The implications of the pandemic, and country-specific responses to the use of vaccine mandates and accompanying digital passes, provides an opportunity to examine trends in online illicit markets and emerging markets for entirely new fraudulent products. In particular, this study explores how an *alt-tech* platform, *Gab*, catered to an online illicit network where fake Australian vaccine certificates were distributed. Alt-tech platforms have largely eschewed the focus of criminologists, despite some initial evidence regarding these digital spaces as harbouring extremist communities (Askanius and Keller 2021; Nouri et al. 2020; Rieger et al. 2021). In the following section, this paper provides an overview of the alt-tech social media platform *Gab* and situates this particular digital environment within the broader digital ecosystem, noting the increasing number of online spaces not easily characterised by binary notions of the ‘dark’ or ‘clear’ web (Copland 2021). Focusing on the distribution of fake vaccine passes, it is therefore important to understand the unique affordances of diversified digital environments as enabling or constraining particular cybercrimes. Although the trade in fake vaccine certificates was temporary, this paper provides evidence for how this trend could be indicative for future developments in illicit networks, including how they assemble in evolving online spaces and distribute illicit products and services.

## Gab as a ‘dark platform’ facilitating the distribution of fake vaccine certificates

This study examines the supply of fake vaccine certificates over the alt-tech social media platform *Gab*^2^. An ‘alt-tech’ platform refers to the collection of social media platforms (e.g. Gab, Truth Social, Parler) that present an alternative option to the mainstream social networking spaces such as Twitter and Facebook, that have purportedly become popular among far-right actors and other fringe online subcultures (Ganesh and Bright 2020; Rieger et al. 2021). The users on *Gab*, in particular, are typically characterised as those who espouse anti-government and anti-regulatory efforts of online spaces. Indeed, the *Gab* community is built on the basis of “*technosocial persecution*” (Jasser et al. 2021) due to how *Gab* openly welcomed individuals banned or suspended from other social media platforms for violating terms of service^3^. The rise of alt-tech social media is also part of the broader development of dark platforms (e.g. 4chan and 8kun) on the Internet, that is, platforms that largely abstain from content moderation and regulation (Zeng and Schafer 2021). Collectively, this development of digital spaces highlights the need for cybercrime researchers to engage with the ongoing diversification of online spaces and the subcultures within these platforms.

### How differentiated digital platforms afford cybercrime activity

*Gab* therefore provides an interesting setting to explore as it occupies a unique space in the internet ecosystem. Although it exists on the ‘surface’ web (the visible portion of the web accessible to all users), its technological underpinnings (e.g. lack of moderation, existing in an ‘alternative’ portion of the web) resemble features commonly associated with the ‘dark’ web. Copland (2021) discusses this increasing complexity of digital spaces with reference to the website *Reddit*, discussing how *Reddit*’s technological changes over time now has it occupying a ‘brackish’ space on the Internet. Studying specific platforms, such as *Gab* in this study, can help to develop an understanding of the technological differences of digital spaces and how this shapes cybercrime activity on online platforms (see Childs et al. 2020; Goldsmith and Brewer 2015; Goldsmith and Wall 2022).

The *affordances* of technologies provides a useful framework for explicating on the technological differences between platforms, including how specific features of platforms shape behaviours of online illicit networks. By exploring how the features of technologies affect the enabling or constraining of human behaviours, affordance-thinking advocates a median ground between technological determinism and constructivism by shifting focus on the agency of technology (Hutchby 2001; Nagy and Neff 2015; Schmidt 2007). Rather than technology wholly determining action, affordance-thinking helps us understand how actions via technologies are made real through technologies and actors assembling in specific conditions (Anderson and Robey 2017; Nagy and Neff 2015). Therefore, rather than homogenising digital spaces where illicit networks develop and the distribution of illicit products occurs, this study recognises online spaces as differentiated by their technological affordances.

## The current study

This study provides the first academic research into the illicit distribution of fake vaccine certificates, focusing on how an alt-tech social media platform (*Gab*) became an exchange space for this illicit product. Exploring this illicit product distribution through *Gab* can provide insights into the unique affordances of emerging digital spaces and how technologies afford cybercrime activities. In doing so, this research contributes to a wider body of criminological research exploring the impacts of COVID-19. While the first wave of research at the intersection of criminology and COVID-19 was primarily interested in the effects of lockdowns and crime trends during lockdowns (Ashby 2020; Boman and Mowen 2021; Murphy et al. 2020), this research engages with cybercrime evolutions during COVID-19 and explores some of the enduring crime problems from COVID-19 as the world shifts to an endemic stage. In the following section I outline the methodological approach for this study on the distribution of fake Australian covid vaccination passes over *Gab*, and in doing so, reflect on some of the opportunities and challenges associated with data collection from online illicit communities. The results from the thematic analysis – including the motivations and ideologies underpinning this cybercrime service, the cybercultural aspects of this online illicit network, and the information sharing on risk reduction strategies – are then described before concluding with considerations on the legacies for this illicit network for illicit markets and cyber organised crime more broadly.

## Method

The proliferation and expansion of online spaces have provided a multitude of new opportunities for data collection (Salmons 2016). This has been particularly valuable in providing access to research populations that have historically been ‘hard-to-reach’ because of perceptions that members outside of their community are threatening and/or because of their own engagement in behaviours that are socially stigmatised or illegal. The use of web data, most often collected in the form of text-based data from forum discussions and marketplace listings, have thus become a vital method for studying online illicit networks and the development of illicit markets on the Internet (Decary-Hetu and Aldridge 2015; Holt 2015). Reflecting on this increased use of text data for understanding crime and online communities, Dupont and Lusthaus (2022) state how “*one of the major challenges faced by social scientists who research cybercrime is access to data that are both sufficiently comprehensive and accurately reflect the social dynamics of a shadowy community of individuals engaging in illicit activities”*. In this context there are some limitations of relying solely on text data from an online community: insight is only ever provided on one aspect of the overall *market* for this product (i.e. there is no information on where or how the hackers/distributors organise themselves) and a number of factors can shape the willingness to contribute to *Gab* posts and share experiences as opposed to simply *lurking* on a platform. Given some of the limitations of relying solely on text-based data from online interactions researchers have also begun assembling multiple ‘digital’ methods together to better examine the features and nuances of illicit networks and services (see Henry and Flynn 2019).

The data collection for this study consisted of two complementary phases. In the first phase, the purpose was to identify fake vaccine distributors on *Gab* via relevant search terms on *Gab*’s search feature (e.g. “COVID pass”). As part of this identification phase there was a brief (1 month) period of digital ethnography (Kozinets 2019), which was helpful for identifying the broader networks of fake vaccine distributors, such as those who ‘shared’ and ‘liked’ relevant posts on *Gab* and the practices of the community. This phase was reflective of ‘best-practice’ research in studying online communities, where the researcher spends time familiarising themselves with the platform and the users including discourses (Kozinets 2019; Wang and Liu 2021). Related to the above discussion on the limitations of solely relying on text-based data, this brief period of digital ethnography was beneficial for examining the platform architecture (Henry and Flynn 2019) and the use of the alt-tech platform *Gab* beyond the confines of this specific context. The intention of this method was not to identify all of the possible fake vaccine certificate distributors over *Gab*, but rather, to provide an indicative account of the nature of this illicit network (including distributors and customers) and how this online space facilitates the distribution of fake vaccine certificates.

Overall, five Australian fake vaccine distributors were identified and included in this study. The distributors of this illicit product are best described as a team of individuals rather than solely as independent operators, as it was evident how the network appeared to distribute tasks and rely on each other to perform hacking tasks. Even when the fake vaccine pass distributors appeared to operate independently (during the data collection one distributor stopped updating the community with developments and seemed to no longer be in contact with the other distributors), this network still resembled a form of distributed organisation (Wall 2015), as there was still the identification and affiliation of the shared goal amongst this illicit service. The social media presence of each distributor are outlined in Table 1. While it is a limitation of this research that the data are not able to quantify the specific number of individuals using fake vaccine certificates, the data still provides an indication of the popularity of this service among community members.

**Table 1:** An Overview of the Presence of Australian Fake Vaccine Distributors on Gab

Distributor	Member Since	Followers	Total Posts and Comments on Profile
D1 D2 D3 D4 D5	October 2021 December 2021 January 2022 January 2022 February 2022	7,000* 326 380 216 54	2081 71 224 193 20

*Gab rounds the follower count to the nearest 100 for accounts with over 1000 followers.

The next phase of this study consisted of the use of a webscraping tool, SimpleScraper^4^, to scrape the posting history of distributors. This involved scraping all text from the original posts from distributors, as well as the comments from community members on *Gab*. The final dataset consisted of 2589 unique comments from distributors and community members of the *Gab* fake vaccine certificate distribution network, which took place in a time period spanning from the 17th of October, 2021–14th of March, 2022. After all comments and posts were scraped the dataset was cleaned (e.g. anonymising posters, removing advertisements) and uploaded into NVivo20 to assist with analysis.

Thematic analysis (Braun and Clarke 2006) was employed on the scraped discussion posts. Initial notes were developed after a period of familiarisation with the discussion posts, by reading through each post line-by-line. Codes were then developed as part of an iterative process by returning to original notes as further meaning was developed and refined. Themes were then developed by combining and/or collapsing the codes from the previous stage of analysis. A combination of inductive and deductive processes were utilised during the coding and theme development of the data in order to allow for novel themes to emerge while simultaneously recognising that researchers are rarely a *blank slate* when analysing data (Braun and Clarke 2018; Terry et al. 2017). Although the posting data were treated line-by-line, this study was also interested in “*observing the natural unfolding of events*” (Miller and Miller 2015) by attending to how narratives changed throughout discussions. Comments quoted in the results section of this paper have been slightly edited for clarity and meaning as well as to apply an additional anonymising mechanism for posters. Finally, where it was appropriate, a conscious effort was made to represent posts across the entire dataset of distributors and their community members.

### Ethical considerations for criminological research on web data

Online data collection on criminal populations raises several ethical principles and safeguarding practices for both participants and researchers: the nature of web data being ‘public’ or ‘private’ and its implication on the requirement of informed consent, as well as the strategies that can be implemented to protect researchers and the population under investigation. Overwhelmingly, criminological research that involves unobtrusive research methods on digital communities has provided a valuable source of data without directly impacting the online community under investigation (Brewer et al. 2021; Martin 2021). When dealing with criminological research in online spaces, it may also be justified to remain hidden as a researcher, as the risks to researchers can potentially increase if they choose to disclose their identity in particular communities (Barratt and Maddox 2016; Martin 2021; Massanari 2018). This was relevant in this context for studying a group inhabiting a digital space typically occupied by far-right groups who have been knowing for threatening and harassing researchers (Conway 2021). Following the guidelines from other researchers facing a similar quandary about the nature of data being public or private (Martin 2021), the community under investigation was deemed as essentially public in nature, as there were no specialised technologies required to access this community, no gatekeeping applied to enter or read conversations in the community, and no attempt to hide posts or distributor profiles. For this research, only publicly accessible information was collected and thus this research carried negligible risk to those involved. In addition, to further minimise the risks to participants, no identifying information (e.g. names, personal details shared in posts) was recorded in the collected posts. Ethics was approved for this research by Griffith University’s Human Research Ethics Committee (GU 2022/105).

## Findings

There are three main categories presented in the findings section that focus on the resulting themes in the data. These themes include: the organisation of this service on the alt-tech platform *Gab*, the cybercultural aspects of this illicit network, and perceptions of the detection and evasion of law enforcement (and other guardians) when using fake vaccine passes in offline settings.

### Overview of digital pass distribution on *Gab*

The first theme captures the dynamics and organisation of this illicit network operating in a unique online space, including emergent motivations for engaging in the use of fake vaccine passports. The alt-tech platform *Gab* was primarily used by distributors to share external links to websites hosting the fake digital passes, whereby the fake digital vaccine passes could be downloaded onto a mobile phone and used in an offline setting where required. The digital ethnography and analysis of discussion posts indicate that this service is characterised as a space where access to the illicit product is temporary and unstable, as there were consistent fluctuations in the availability of this service and prolonged periods where these services could not be accessed. The most frequent comments from community members were in relation to the website being unavailable and requests for the distributors to fix the website for accessibility to fake vaccine certificates.

Engaging with the history of posts over the lifetime of the *Gab* distributors also provides some evidence of the evolution of this illicit service over time. Rather than performing a service solely for fake vaccine certificates, the distributors were reflexive to changing public health policies and customer demand. For instance, while initially the distributors focussed solely on developing phone apps that displayed a fake vaccine certificate, this service developed over time to offering a wider range of services such as fraudulent border passes to enable individuals to move between state borders in Australia. The implementation of new public health directives and laws were arguably creating the conditions for further development of services in this illicit network (Jaros 2012). In addition, although the distributors operated primarily for an Australian community, these services also evolved to developing products beyond an Australian context. As the popularity of the distributors increased because of their technical prowess in making fake vaccine certificates, there was an increasing sense of extraterritoriality when it came to the services on offer, as they increasingly responded to requests to develop a range of fake services for other countries including the United Kingdom, New Zealand, and Australia where similar public health requirements for implemented during COVID-19:

*Hey is anyone you know making UK versions and UK vax certificates?* [Distributor reply]: *I could*

There was indication that the Gab distributors represented only a fraction of the overall organised efforts to crack digital vaccine passports around the world:

*…collective effort has gone into cracking Israel and Italy’s ones first as they are most severely oppressed.*

The fake vaccine certificates (and other services) available through *Gab* distributors were provided at no cost to those wishing to use the services. However, donations in the form of cryptocurrencies (e.g. Bitcoin, Monero, and Ethereum) were accepted and often advertised as a way to help support the distributors in their efforts to keep the service available and fund further tools. Occasionally, the use of cryptocurrency donations was a way to incentivise the distributors to accept certain jobs and work on updates for the services (“*Is there anyone out there who can mimic the South Australian app? Happy to make a donation that covers time”*). The fact that this illicit service operated free-of-charge, as opposed to typical cybercrime markets and services available on the web (Liggett et al. 2020), provides insight into the ideologies and motivations for this cybercrime service. This illicit online service was not assembled as a space where multiple vendors are financially-motivated and competing for customers, but rather, this was an online community where the distribution and use of the product was motivated by various ideologies.

### Motivations for distributing and using fake vaccine passes

The analysis of discussions on *Gab* posts revealed some of the key motivations and ideological aspects of the distribution of fake vaccine certificates, including why distributors were willing to provide a service free-of-charge. The criminological framing of illicit markets is typically one characterised by profit-seeking actors, but this is not always on display when exploring various dimensions of the illegal supply of goods and services (Bancroft 2019; Childs et al. 2020a, b, c). For example, in illicit drug supply, the ‘*social supply*’ of drugs (see Coomber and Moyle 2014) is often performed in contexts where drugs are provided free-of-charge or without the suppliers opting to take a profit, as instead, the exchange facilitates friendship connections and resembles gift-giving behaviours. In the cybercrime environment, ‘hacktivists’ have also historically eschewed seeking profit from hacking activities because of the underlying political and ideological motivations. The distributors were clearly motivated to supply the illicit product to enable a grander community goal of freedom from government interference, even if that meant that services were provided free-of-charge despite significant time investment for their work. Libertarian discourses permeated reasons for engaging with the illicit COVID-19 services, as it enabled individuals to have their “*freedom*” restored: to remain employed at their occupation, to visit family members in hospital, and visit retail stores, bars, and restaurants unencumbered.

Perhaps unsurprisingly, anti-vaccination views were a common set of beliefs expressed by individuals on this platform. Often permeating narratives of anti-vaccination beliefs were notions of conspiratorial thinking in relation to government-mandates in personal health choices as being part of a broader agenda:

*The globalists and cabals are losing big time...like decades of plans, investments, time are being destroyed in some cases in as little as 4 weeks. We are going to struggle and suffer for a bit longer and it will get worse before it gets better but let me tell you with absolute truth that the world we will inherit on the other side of this mess will be a future brighter than we could have possibly imagined. You need to get as many people on Gab as possible. This is the community leading the fightback against the Anti Christ and global tyranny.*

*I do not want to be their fucking cobaye. I do not want their great reset. I choose to be free to travel where I want without being tracked. I choose what I will or will not inject myself with. I have sole sovereignty over my body and my mind. I choose Freedom. And they have no say.*

Reflected in these quotes were the beliefs from some community members that the public have been lied to about the true purpose of vaccine mandates, and by engaging in the use of fake vaccine passes they were an important community leading a resistance against the government. Online spaces, and alt-tech platforms such as *Gab* in particular, have had a tendency of nurturing conspiracy theories (Forberg 2021; Mahl et al. 2022), and the results in this study extend on our understanding of the consequences of platforms fostering conspiracy theories by way of providing motivations to engage in crime. Importantly, this study also found that such conspiratorial thinking was not unconditionally accepted by community members (Stano 2020) as some users challenged the beliefs of those spreading conspiracy theories.

Finally, even for those who did not accept conspiratorial claims related to vaccine mandates, the expression of fears relating to surveillance were a considerable motivator for using these illicit vaccine passes. Both state and corporate surveillance (although it is increasingly difficult to make distinctions in the context of surveillance) has increased significantly over the last two decades, whereby contemporary society is best characterised as a ‘surveillance society’ as lives become subject to ever-expanding means of surveillance (Lyon 2001). The increase in surveillance mechanisms is also a product of responses to significant events (e.g. 9/11 terrorist attacks) (Innes 2001) which are also reflected in the current context. There has undoubtedly been increases in state/corporate surveillance during the COVID-19 pandemic (Bernot et al. 2021; Eck and Hatz 2020; Lyon 2021), and the results from this study show how illicit markets developed in this context as a response to individuals’ perceived needs for anti-surveillance measures. Here, the use of a fake vaccine passport to minimise an individual’s digital footprint was amongst a wide array of other privacy-preserving tools that community members discussed as being a part of their daily life (e.g. cryptocurrencies, Brave Browser, Duck Duck Go, Telegram, and encrypted mail services such as Proton Mail).

### Cybercultures of illicit networks: customer service in cybercrime markets and innovative crowdsourcing processes

The second theme discussed herein refers to the cybercultural aspects of this illicit network. Building a sense of ‘community’ has become a critical component of spaces where cybercrime services are available (Holt and Dupont 2018; Ladegaard 2019). On *Gab*, the affordances of ‘liking’ and ‘commenting’ on posts encourage social interaction and connection between individuals inhabiting the same online space. Developing a community is also partly a product of the broader recognition of “*cybercrime as a service*”, the process of deviant subcultures transitioning to the maintenance of tools and customer service as priorities (Collier et al. 2021; Manky 2013). In many contemporary online illicit markets, customers seek satisfactory experiences and speedy responses from vendors when issues arise (e.g. the unsuccessful delivery of a product), and successful operators are generally those who are customer-focussed, can build resilient communities, and idealise efficient customer service as part of their trade (Bakken et al. 2018; Holt 2013). This was also evident in the *Gab* community, whereby responses to technical requests from community members (e.g. the hosting website was unavailable, assistance with technological challenges, and vendors providing software update logs) was frequently witnessed. In contrast to wider cybercrime research, an ethos of customer service and building communities is not evident solely in markets where economic competition between vendors reinforces the need for customer service and trust building processes, but it is also evident in smaller illicit networks where services are provided free-of-charge.

This accepted use of the online community as a hub for assisting with technical problems was not always a defining feature of this community. Over time, as the service became more popular with users, the willingness to engage in a customer service role diminished. In the following posts from a distributor, the importance of self-reliance in the community when engaging in technologically facilitated crimes came to be emphasised:

*If there are boomers and shit who don’t know what to do HELP THEM! If you are technologically savvy help out those who are not!*

*Here is some resources for those who don’t know how to install our apple ios apps: [link]. We can not provide any help for this as we get swamped with thousands of emails and messages a day about it. You need to learn how to do these things yourself so you can learn tech skills. If you become dependent on us you will never grow and become stronger.*

As the size of the network increased there was also a budding need for end-users of the service to empower themselves in technical matters for accessing and navigating illicit services in this digital space. In a similar manner, Kowalski et al. (2019) illustrates how self-reliance is a critical feature of drug buying over dark web drug cryptomarkets, and that “*…learning how to use a cryptomarket is then left to the coordination, discretion and direction of the interested individual, in the absence of an overarching coordinated socialising effort”*. There are similarities between these different forms of digital crimes, that also take place over disparate technological landscapes, that provides further evidence for how the political and philosophical underpinnings in the design of technologies shape behaviours (Miller 2020; Shaw 2017). In this context, the political ideologies underpinning the creation of *Gab* – libertarian values – can be seen to shape both the behaviour evident on the platform (i.e. illicit tools that oppose government mandates) and shifts in the ethos of the community surrounding this distribution (e.g. self-reliance).

An innovative aspect of this network, largely facilitated by technological affordances enabling rapid communication and the ability to share photos and videos, was the way distributors engaged in crowdsourcing processes in order to build the most effective fake vaccine passes. Crowdsourcing is generally defined as the process involved when organisations leverage large swathes of information from online communities to benefit particular tasks or the development of products (Brabham 2012). Although criminological research has explored the role of crowdsourcing in the context of Facebook pages where community members post the locations of policing activity (Wood and Thompson 2018), the *Gab* distribution network for fake vaccine passes demonstrated dynamic top-down and bottom-up processes for collectively building illicit digital tools. Bottom-up processes of crowdsourcing was the most common practice, whereby community members would often comment on posts making suggestions to improve the authenticity and perceived legitimacy of fake vaccine passes:

*Just an fyi on the printable certs, the coat of arms is way too high. The text at the bottom is a little off as well in comparison to the real one.*

*Other than the animation of the hologram looking like it’s been sped up x300 to how a normal person would hold a phone, great job dude, ahaha! … I dmed you a video that shows how slow the movement is. If you need more let me know. Also hologram/coat of arms need to be slightly bigger as well.*

The selected quotes highlight how information from community members in crowdsourcing spaces is an effective means to gather ideas and solutions to improve services (Wexler 2010). By engaging in crowdsourcing processes, community members receiving the fake vaccine passes are no longer passive recipients of an illicit end-product, but rather, the community are an innovator and problem solver, along with the distributors, in co-creating the fake digital vaccine certificates. In contrast, top-down processes of crowdsourcing were also on display, as reflected in various distributor posts making specific requests for information:

*If you are in Western Australia and have ServiceWA, it would be really helpful if you could send me through screenshots of what each of the 5 app tabs look like when you click them. In addition to the vax pass and check in, I want to put in a mockup of the contents of the “Services”, “G2G” and “Inbox” screen so if someone starts to click around on there it just looks like the normal app. If there’s identifying info on the screens then do redact it before sending through. The screenshots will be also important because of the icons on the menu bar, they light up from grey to white when the tab is selected so I need to extract copies of each of those icons so I can add them to the fake app.*

*Need photos and/or videos of the checking in process for South Australia. I do have one screenshot of the “checked in” confirmation screen and of the vaccine passport, both from the app store, but what I would like to see is the screen when you first open the app, and of course the sequence of steps after directly scanning a QR code, e.g. “do you want to add any dependants”, so I know precisely how the app needs to look for replication. I even have some demo code courtesy of @[Distributor 2] which reads the location name automatically from the QR code.*

Top-down crowdsourcing processes recognise that community members will not always be rationally motivated to contribute to discussions. Therefore, by setting agendas for discussion and detailing specific tasks for community members, there is an incentive to participate in discussions and contribute information (Childs et al. 2020a, b, c; Ren et al. 2019). Technologies that allow for collaboration, by affording particular behaviours (e.g. sharing images of legitimate vaccine passes to replicate), were essential in these crowdsourcing processes for illicit tools.

The results from this study also highlight how the *Gab* fake vaccine certificate community intersected with other digital spaces. This intersection with other online spaces and digital communities was reflected in the concept of multichannel retailing in online illicit markets (Childs et al. 2020a, b, c). In multichannel marketing for illicit services, vendors will establish multiple points of communication with prospective customers akin to legal retailers that simultaneously operate across many physical/digital platforms simultaneously (Dholakia et al. 2010). Telegram (an encrypted messaging application) channels, for example, were offered as another digital space for customers to access the services on offer and chat to other community members. These changes across technology platforms offer varied affordances. In this instance, distributors have the opportunity to diversify customer interactions and capture a new market segmentation on an alternate digital platform. However, it is also critical to recognise how the active sharing of group details, and encouraging the community members to share information widely about how these tools could be accessed, is a balancing act in the maintenance of illicit networks. Although small groups can ensure the viability of an illicit network (Fabiani and Behlendorf 2021; Morselli 2010), small groups also limit the financial opportunities for distributors (e.g. cryptocurrency donations for their services) and constrains ideological motivations for creating the product (e.g. building tools to empower individuals to subvert government mandates). However, sharing group details too widely can result in increased law enforcement efforts and media attention, and thus attempts to counteract this illicit service. The final theme exploring the perception, detection, and evasion of law enforcement (and other relevant guardians) is elaborated on in the following section.

### Perception, detection, and evasion of guardians

A critical function of the *Gab* network was to gather community members’ perceptions of risk and provide tips for evading guardians when using fake vaccine passports. These discussions were predominately centred on specifying the appropriate contexts of use for successfully using a fake vaccine pass. The risk of using fake vaccine certificates was shaped by the varying contexts of use. Settings such as workplaces, universities, bars, and domestic travel purposes (e.g. airlines, state border crossings) were generally perceived as low-risk examples whereby fake vaccine passes could be used without any personal or legal ramification. International travel and settings that would require an interaction with government databases were generally seen as high-risk examples that should be avoided. Users shared their own experiences of successfully using fake vaccine passes and responded to other community members’ anxieties about using fake vaccine passes in attempts to assuage feelings of risk. Negative experiences were also shared amongst the community members as a way to demonstrate some of the faults in the service and how to evade law enforcement and other guardians:

*I got pulled up by a guy at check in at a cafe and he knew it was fake, but only because the green bar on the first page is not as wide as it is on the proper app … He let me in anyway, because he was a good guy and didn’t care…*

*I’ve been using the fake one for over a week now and honestly it has not been scrutinised once, I highly doubt such a minor detail would immediately flag the app as a fake like you’ve stated considering the wide range of devices being used by the population and so many folks don’t even know there are fakes, there’s going to be some noticeable differences in appearance of the screens… IMHO it all comes down to the delivery... Sell it like a used car salesman sells lemons, Confidence is key and have the fake medicare app loaded as well as a backup, no one’s gonna argue with you over its legitimacy.*

*Sorry it wasn’t my intention to scare anyone. Just that my husband used your link for the app and got caught at the gym because they recognized the document number. I’m assuming it’s because people think this every number is different for everyone and they don’t change it…* [Community member reply]: *Why didn’t he change it? Rookie.*

Public health orders mandating vaccination status at retail outlets, cafes, bars, and restaurants often emphasised the role of employed staff as a guardian (Hollis-Peel et al. 2011) for preventing violations. For the users of fake vaccine passes, evading the detection of guardians at establishments, as seen in the quoted posts above, was a socio-technical process assembling the forces of humans and technologies. The technical details of the fake vaccine passes were essential (e.g. the correct placement of logos, fonts, and security features) as was the individual’s role in knowing the appropriate settings for use, ensuring that personal details are correctly entered into templates, and having confidence when approaching venue guardians tasked with checking the vaccination status of patrons.

Although the use of fake vaccine passes would occur mostly in physical spaces, the community members were also aware of the datafication of their illicit activities and how to minimise their own digital traces that could detail involvement in this illicit service. Online activities inevitably result in digital traces, as interactions with digital platforms and tools are performed amongst mass data collection efforts from platform providers and services. More specifically, this quantification of online activities has been key to business models as data itself has become a form of capital (Sadowski 2019). The power of data has also creeped into the imaginaries of ‘smart’ criminal justice system technologies, whereby, the use of Big Data and digital traces can become fundamental to the process of detecting and responding to digitally-enabled crimes (Lavorgna and Ugwudike 2021; Smith et al. 2017). For example, in this context, one distributor was often warning others about how the security features of Apple’s iPhones could expose their activity, suggesting how although Android branded smartphones are “*still shitty big tech*” they were “*way less intrusive than iPhones*”. Illicit networks in online spaces therefore recognise the power of their own digital traces and that data is not equal in its capacity to increase risks related to law enforcement, as some companies and service providers are more willing to share data with law enforcement and cooperate on legal matters than others.

## Concluding thoughts: the legacies of fake vaccine certificate distribution

This paper provides the first exploration of the distribution networks that have emerged for fake COVID-19 vaccine certificates. By focusing on the alt-tech platform *Gab*, this paper explores the mechanics behind an online illicit network that developed for distributing fake vaccine passes in an Australian context. This study highlights the various affordances of *Gab* that fostered this distribution: the ability to share links to external sites hosting illicit products and services, the accessibility of this social media platform, the lack of content moderation, and the cultivation of a community with binding ideologies for using and distributing fake vaccine passes. In doing so, this study revealed evolutions in the way digital spaces are used for the distribution of illicit products, noting how these tools were a function of crowdsourcing practices, and how this community used *Gab* to evade law enforcement detection through sharing experiences in this online space.

It is worth reflecting on the legacies and future implications of this illicit network that developed during COVID-19. With the continued relaxation of vaccine mandates worldwide, this illicit service will at some point become completely defunct, but these findings have various implications beyond COVID-19. In many respects COVID-19 has had lasting effects that have persisted even following lockdown orders (e.g. transforming work practices and shopping habits) (Florida et al. 2021). In this context, the rapid growth of this illicit service for fake vaccine passes raises several potential cybersecurity concerns and risks surrounding the implementation and use of digital identification systems, as well as organised crime trends that respond to the implementations of such technologies. Many countries and regions around the world, including Australia, Ireland, Canada, and the European Union are currently in the early stages (including trials) for implementing digital identification systems (Cater 2021; Macdonald 2022; McNamee, 2022). These systems often involve the digitalisation of identities and centralised access to government services. This research has highlighted the ease at which hacking collectives (however loosely organised) can develop tools to counteract government requirements, the ease at which these communities can recognise and implement security features (e.g. holograms) to counterfeit digital documents, and how online spaces will develop to distribute digital documents.

Though there is some debate about the extent to which many cybercrimes can be usefully understood as forms of organised crime (Leukfeldt et al. 2017; Wall 2015), this study explored how a loosely organised group of fake vaccine distributors adopted an online platform to distribute their service. This has further implications for understanding the evolution of illicit markets and networks in online spaces. By adopting the frameworks in affordance-thinking and attending to the unique features of digital platforms where illicit markets and communities are developing, cybercrime research and organised crime research exploring digital platforms more broadly can move beyond depictions of digital environments as a homogenised space where crime and networks emerge. This is critical for developing appropriate prevention solutions, which will be dependent upon understanding the unique aspects of digital spaces in affording illicit market practices.

## Footnotes

1 At the time of writing, in September 2022, almost all vaccine mandates have been removed.

2 https://gab.com.

3 Controversial figures, such as Alex Jones, were welcomed on Gab following their bans on other popular social media platforms.

4 SimpleScraper (https://simplescraper.io) is Google Chrome plug-in for scraping websites.
